# Obesityand Gynaecological Cancers, with a Focus on Morbid Obesity: Risk Stratification, Early Diagnosis and Management

**DOI:** 10.3390/diagnostics16142295

**Published:** 2026-07-22

**Authors:** Magdalena Bizoń, Karolina Piotrowska-Lis, Anna Sztokinier, Justyna Domienik-Karłowicz, Maciej Olszewski, Anna Rulkiewicz

**Affiliations:** 1 LUX MED Oncology Hospital, ul. Szamocka 6, 01-748 Warsaw, Poland; magdalena.bizon@luxmed.pl (M.B.); karolina.piotrowska-lis@luxmed.pl (K.P.-L.); anna.sztokinier@luxmed.pl (A.S.); maciej.olszewski@luxmed.pl (M.O.); 2 Wyższa Szkoła Nauk Medycznych LUX MED, ul. Szturmowa 2, 02-678 Warsaw, Poland; 3 Public Health Care Centre, Blessed Virgin Mary Hospital, ul. Starowiejska 15, 08-110 Siedlce, Poland; 4 LUX MED Group Ltd., 02-678 Warsaw, Poland; anna.rulkiewicz@luxmed.pl

**Keywords:** obesity, morbid obesity, gynaecological cancers, endometrial cancer, ovarian cancer, cervical cancer, prehabilitation, oncology

## Abstract

Obesity is a chronic, relapsing disease and a significant oncological risk factor. The correlation is most pronounced and consistent for endometrial cancer. Conversely, evidence linking obesity to ovarian cancer is less robust and varies by histotype, while the association with cervical cancer is influenced by factors related to screening, diagnosis, treatment, and survival. This review examines obesity, particularly class III (morbid) obesity, in relation to the risk of gynaecological cancer, diagnostic approaches, and management strategies. A structured narrative review of PubMed/MEDLINE, Cochrane Library, Scopus and Web of Science Core Collection was conducted for literature published between January 2000 and December 2025. Eligible evidence included systematic reviews, meta-analyses, cohort and case–control studies, mechanistic studies and clinical guidance relevant to obesity and endometrial, ovarian or cervical cancer. Title/abstract screening and full-text selection were conducted using predefined criteria for conceptual relevance and clinical applicability. Excess adiposity contributes to endometrial carcinogenesis through hormonal dysregulation, insulin resistance and hyperinsulinaemia, adipokine imbalance, chronic inflammation, and oxidative stress. In ovarian cancer, associations are generally weaker but appear more relevant for selected histological subtypes and cumulative adiposity exposure. In cervical cancer, obesity should not be interpreted as replacing HPV-driven pathogenesis; rather, it may affect screening adequacy, treatment selection, perioperative risk, and disease-specific survival in morbidly obese patients. Current evidence does not support morbid obesity as an independent driver of all gynaecological cancers. It supports obesity as a major modifiable risk factor and clinical modifier, particularly for endometrial cancer, and highlights the need for pragmatic risk stratification based on BMI class, adiposity distribution, metabolic comorbidity, functional status and cancer-site-specific pathways. Biomarker evidence remains hypothesis-generating, and obesity-integrated oncological pathways require prospective validation in patients with a BMI ≥ 40 kg/m^2^.

## 1. Introduction

Obesity is a chronic, relapsing and progressive disease that has been recognised by the World Health Organisation as a global clinical problem over the last few decades. Its prevalence has nearly tripled worldwide since 1975, and current estimates indicate that more than 650 million adults are living with obesity [1]. The condition is now understood not merely as an excess of body weight but as a complex metabolic and inflammatory disorder arising from interactions among genetic susceptibility, environmental exposures, behavioural factors, and endocrine dysregulation. Consequently, contemporary management strategies increasingly focus on comprehensive, long-term interventions, including structured lifestyle modification, pharmacotherapy (e.g., GLP-1 receptor agonists and dual incretin therapies), and metabolic bariatric surgery, which have demonstrated substantial weight reduction and improvement in obesity-related comorbidities [2].

Importantly, accumulating evidence indicates that intentional weight loss may reduce cancer risk and improve oncological outcomes. Observational studies of patients undergoing metabolic bariatric surgery show a significant reduction in overall cancer incidence, particularly for hormone-dependent malignancies, compared with matched obese controls. Pharmacological weight-reduction therapies have also shown beneficial effects on metabolic and inflammatory parameters implicated in carcinogenesis, although long-term oncological outcome data remain limited [3,4].

**Figure 1:**
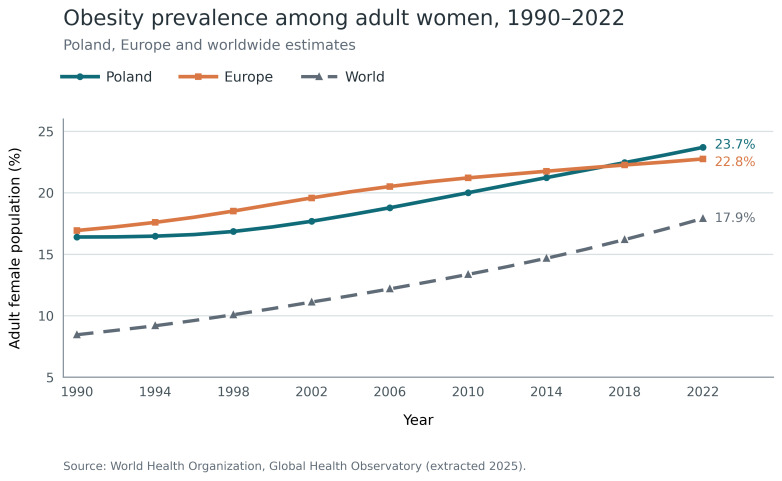
Prevalence of obesity among adult women in Poland, Europe and worldwide, 1990–2022. Values are presented as percentages of the adult female population—data source: World Health Organisation, Global Health Observatory, extracted in 2025.

The global prevalence of obesity has risen substantially in recent decades. According to data from the World Health Organisation Global Health Observatory, obesity prevalence among adult women has continued to rise across Poland, Europe and worldwide, as shown in Figure 1.

Obesity is commonly classified by body mass index (BMI). Higher BMI categories are associated with a greater metabolic burden, a higher prevalence of obesity-related comorbidities, lower quality of life, and more complex diagnostic and treatment pathways. In this manuscript, morbid obesity refers to class III obesity, defined as BMI ≥ 40 kg/m^2^.

Oestrogen-dependent gynaecological cancers may be influenced by aromatisation of androgens to oestrogens in adipose tissue, altered sex-hormone-binding globulin concentrations, insulin resistance, adipokine imbalance, and chronic low-grade inflammation [5].

This narrative review aims to evaluate the evidence linking obesity—specifically morbid obesity (BMI ≥ 40 kg/m^2^)—to endometrial, ovarian and cervical cancers and to distinguish between cancer incidence, cancer-specific mortality, overall survival, non-cancer-related mortality and treatment-related morbidity. A secondary aim is to propose an obesity-integrated oncological pathway for patients with morbid obesity.

## 2. Methods

This manuscript is a structured narrative review, not a systematic or scoping review. A structured literature search was conducted in PubMed/MEDLINE, the Cochrane Library, Scopus and the Web of Science Core Collection for publications from January 2000 to December 2025. The search combined terms related to adiposity and obesity severity (“obesity”, “severe obesity”, “morbid obesity”, “class III obesity”, “body mass index”, “BMI”, “visceral adiposity”, “waist circumference”, “metabolic syndrome”, “insuline resistance”) with terms related to gynaecological oncology (“endometrial cancer”, “endometrial carcinoma”, “ovarian cancer”, “cervical cancer”, “gynaecological cancer”, “cancer incidence”, “cancer-specific mortality”, “overall survival”, “recurrence”, “perioperative complications”, “prehabilitation”, “bariatric surgery”, “GLP-1 receptor agonist”). Eligible sources included systematic reviews, meta-analyses, large cohort studies, case–control studies, Mendelian randomisation studies, mechanistic studies, clinically relevant translational studies and society guidance addressing obesity and gynaecological cancer risk or management.

Potentially relevant records were first screened at the title and abstract level for relevance to adult gynaecological oncology, obesity/adiposity exposure, cancer-site specificity and clinical applicability. Full texts were then assessed for contribution to one or more predefined evidence domains: incidence/risk, cancer-specific mortality, overall survival, non-cancer-related mortality, treatment-related morbidity, mechanistic plausibility and obesity-specific management implications. The final synthesis comprised 59 cited sources. Because the objective was interpretive and clinically integrative, formal PRISMA screening, duplicate-removal counts, meta-analysis and risk-of-bias scoring were not undertaken.

**Figure 2:**
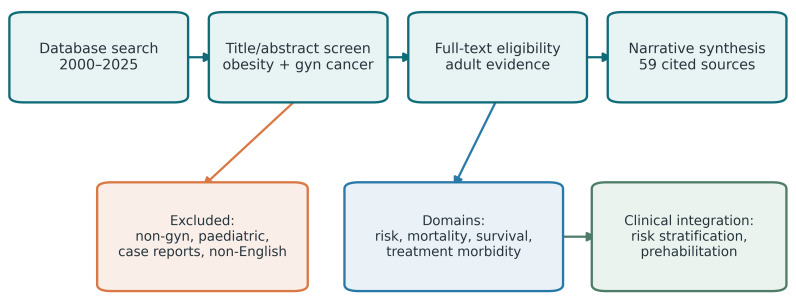
Narrative evidence-selection map. The diagram summarises the structured narrative search and synthesis process. It should not be interpreted as a PRISMA flow diagram, as this review was not designed as a systematic review.

Direct evidence reporting class III obesity (BMI ≥ 40 kg/m^2^) as a separate exposure category was limited. Therefore, the synthesis explicitly differentiates evidence specific to morbid obesity, where available, from broader obesity evidence based on BMI ≥ 30 kg/m^2^, BMI ≥ 35 kg/m^2^, BMI per 5 kg/m^2^ increments, waist circumference, visceral adiposity, or metabolic-syndrome markers. Broader obesity evidence was used to support biological and clinical plausibility but was not treated as direct proof of a class-III-specific effect (Figure 2).

Terminology was standardised throughout the manuscript. “Incidence” refers to new cancer diagnoses; “cancer-specific mortality” to deaths attributed to the malignancy; “overall survival” includes deaths from any cause; “non-cancer-related mortality” to deaths attributable to competing comorbidities or other causes; and “treatment-related morbidity” to complications or adverse events associated with diagnostic, surgical, radiotherapy or systemic treatment pathways.

## 3. Pathophysiology of Obesity

Obesity should be recognised not only as a metabolic disorder but also as a modifiable oncogenic risk factor, particularly for female-specific cancers such as endometrial and ovarian cancers. Understanding the complex biological interplay among adiposity, endocrine disruption, insulin resistance and chronic inflammation is essential to developing effective prevention and therapeutic strategies.

The detrimental impact of obesity on the development and progression of metabolic diseases, including type 2 diabetes mellitus, musculoskeletal disorders, and cardiovascular diseases—with a consequent increase in overall and cardiovascular mortality—is well established. However, growing epidemiological and mechanistic evidence indicates that obesity also significantly increases the risk of numerous malignancies, commonly referred to as obesity-related cancers.

According to the International Agency for Research on Cancer (IARC), excessive adipose tissue is causally associated with at least 13 types of cancer, including several female-specific malignancies. Reported relative risks (RRs) range from approximately 1.1 to over 7.0, depending on cancer type and degree of adiposity, with the strongest association consistently observed for endometrial cancer [1,3,6].

Obesity is characterised by body mass index (BMI) and is classified into the categories shown in Table 1.

## 4. Epidemiology of Gynaecological Cancers

Gynaecological cancers are a group of female-specific neoplastic diseases that include endometrial, ovarian, cervical, vulval and vaginal cancers.

**Figure 3:**
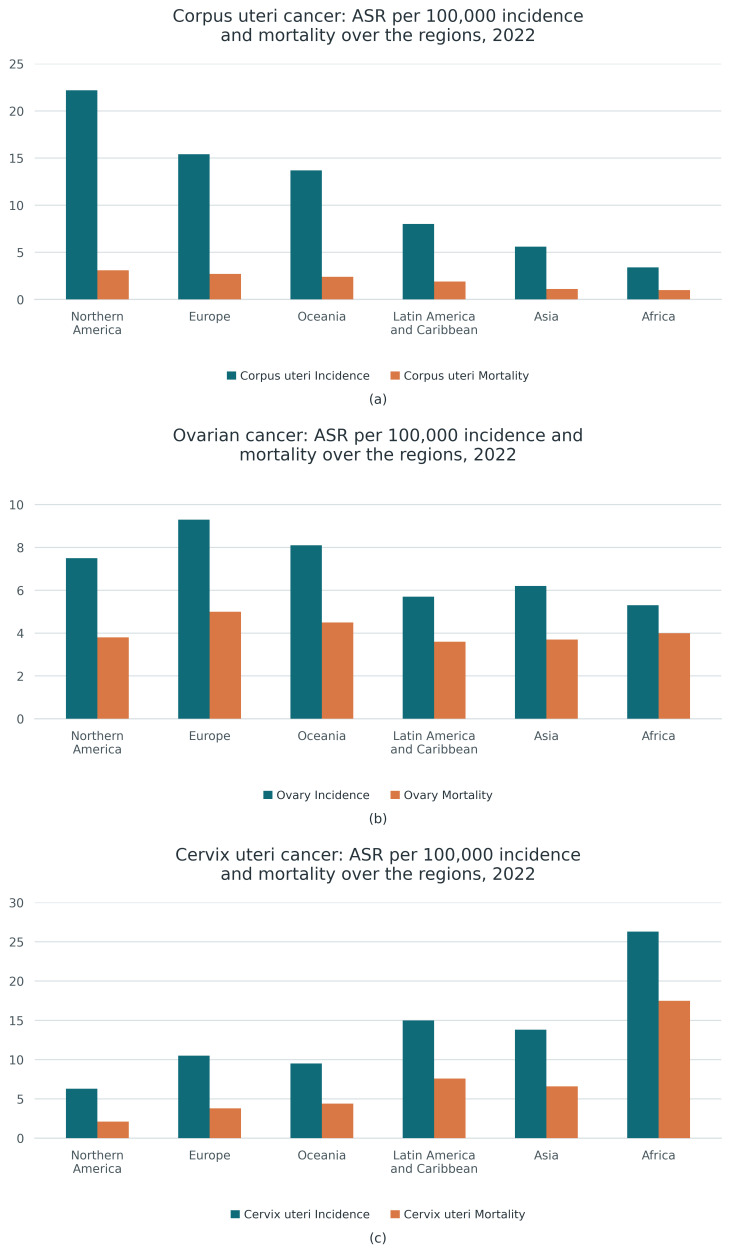
Age-standardised incidence and mortality rates for selected gynaecological cancers in 2022: (**a**) corpus uteri/endometrial cancer; (**b**) ovarian cancer; (**c**) cervical cancer. Rates are presented per 100,000 women. Data source: World Health Organization/International Agency for Research on Cancer, Global Cancer Observatory, 2025 extraction.

Incidence and mortality of endometrial, ovarian and cervical cancers are presented in Figure 3. The epidemiology of these cancers depends on lifestyle, diet and physical activity, environmental factors, genetic mutations, family background and other factors.

Modifying diet and lifestyle can, in most cases, lead to weight loss, enhance well-being, and reduce incidence rates. Increased awareness and understanding of cancer risk factors facilitate early diagnosis of the disease.

## 5. Obesity as a Risk Factor for Gynaecological Cancers

Obesity is characterised by excess adipose tissue accumulation, yet oncological risk is not captured by BMI alone. Central adiposity, visceral fat volume, insulin resistance and cumulative lifetime exposure to excess adiposity may provide additional risk information, particularly for endometrial cancer. The evidence base should therefore distinguish general obesity, visceral adiposity, metabolic syndrome and morbid obesity, rather than treating all BMI categories as equivalent.

For cervical cancer, obesity should be interpreted differently from how it is for endometrial cancer. HPV infection remains the dominant causal factor, whereas obesity may act as a modifier of screening adequacy, diagnostic delay, treatment feasibility and survival rather than as a primary aetiological driver [7,8,9,10].

Zhang et al. analysed a cross-sectional dataset from the National Health and Nutrition Examination Survey (NHANES) conducted between 2007 and 2018. Among 7855 women, 237 were diagnosed with gynaecological cancer, including ovarian, endometrial and cervical cancers.

Higher waist circumference, body mass index, and triglyceride levels were observed in patients with gynaecological cancers. The same group also showed a higher prevalence of metabolic disorders such as hypertension and diabetes. In every case, age-adjusted visceral adiposity index (AVAI) was calculated using data on HDL, WC, BMI, and TG levels. The study found that AVAI was associated with a 28% higher risk of gynaecological cancers. This association was particularly observed in cervical cancer. Other associations with ovarian and endometrial cancers were not statistically significant (*p* > 0.05) [11].

By 2025, approximately 1.2 million women worldwide were expected to be diagnosed with gynaecological cancer [12,13,14]. The rising incidence of endometrial cancer is particularly relevant, as it coincides with increasing obesity prevalence and metabolic disease burden. However, the strength and clinical interpretation of this association vary by cancer site and outcome domain.

Obesity frequently coexists with cardiometabolic, respiratory, renal and thromboembolic comorbidities. These conditions may affect access to diagnosis, anaesthetic risk, surgical approach, radiotherapy planning, systemic treatment dosing and survivorship care; therefore, patients with morbid obesity require cancer-site- and pathway-specific risk stratification. A structured summary of the key evidence is provided in Table 2, and its site-specific clinical interpretation is presented in Table 3.

### 5.1. Endometrial Cancer

#### 5.1.1. Pathophysiology

Endometrial cancer is the gynaecological malignancy most strongly and consistently linked to obesity. Large meta-analyses indicate that each 5 kg/m^2^ increase in BMI is associated with a 50–60% increase in endometrial cancer risk [16,25]. Nevertheless, BMI is an imperfect surrogate for oncogenic adiposity. Adult weight gain, duration of obesity, waist circumference and visceral fat distribution may more accurately capture biologically relevant exposure than a single BMI measurement because visceral adipose tissue functions as an endocrine and inflammatory organ.

The obesity-related endometrial carcinogenic pathway can be systematised into four overlapping domains. First, hormonal dysregulation increases oestrogen bioavailability through aromatisation in adipose tissue and reduced sex-hormone-binding globulin. Second, insulin resistance and hyperinsulinaemia increase insulin-like growth factor signalling and stimulate endometrial proliferation. Third, adipokine imbalance, including hyperleptinaemia and reduced adiponectin signalling, promotes angiogenesis, mitogenic signalling and impaired anti-proliferative control. Fourth, chronic low-grade inflammation and oxidative stress increase cellular stress, mitochondrial reactive oxygen species and DNA damage; in endometrial tissue with mismatch-repair defects, this may facilitate the accumulation of deleterious mutations and progression from hyperplasia to carcinoma [1,25,26,27]. Microbiome dysbiosis has also been proposed as an emerging mechanism linking obesity, inflammation and endometrial carcinogenesis, but this remains less clinically established than the endocrine and insulin-mediated pathways. These domains are summarised in Table 4.

#### 5.1.2. Translational and Biomarker Evidence

Preclinical and translational studies help explain biological plausibility but should not be overinterpreted as direct clinical proof. In a mouse model, Kim et al. investigated the interaction among obesity, leptin deficiency and Pten alteration, reporting more aggressive endometrial tumour biology in the combined obesity/Pten-deficient context [28]. These findings support mechanistic plausibility but do not establish that morbid obesity independently determines human tumour stage.

Similarly, adipocytokine and biomarker studies suggest that obesity-related inflammatory and metabolic signals may be linked to aggressive pathological features, including lymphovascular space invasion, mismatch-repair-deficient subtypes, or deep myometrial invasion [26,27]. These studies are best interpreted as hypothesis-generating and should be distinguished from epidemiological evidence on incidence or survival.

An additional critical component in the pathogenesis of obesity is the adipocytokine group, comprising adipokines and cytokines. Among the most biologically active adipokines, adiponectin contributes to the development of insulin resistance and diabetes. Increased adiponectin expression was observed within the lymphovascular space invasion of the tumour in endometrial cancer. During the initial stages, such detection was not evident [26].

STC-1, adiponectin and galanin have been investigated as potential biomarkers for endometrial cancer and obesity-related metabolic dysfunction [26,27,29]. Their current relevance is mechanistic and exploratory; none of these biomarkers is established or recommended for routine obesity-based risk stratification, early detection or treatment selection in gynaecological oncology.

#### 5.1.3. Impact of Obesity on Quality of Life

Torres et al. conducted a study on patients’ knowledge of the association between endometrial cancer and obesity. Only 11.1% of patients with low-risk endometrial cancer discussed the correlation between obesity and cancer with gynaecologists. The analysed population was divided into a historical cohort without surgical preparation and an interventional cohort that received preoperative consultation and education about obesity. Greater awareness of obesity enables patients to attend a weight-loss clinic and take action to manage their condition [30].

Nock et al. evaluated sleep quality, quality of life and depressive symptoms in 100 endometrial cancer survivors with obesity who were seeking weight loss. The cohort had a mean BMI of 42 kg/m^2^, and nearly 60% had morbid obesity; worse sleep quality and depressive symptoms were common [31]. This finding should not be interpreted as specific to endometrial cancer, as sleep disturbance and depression are also common in obesity and in cancer survivorship more broadly. It does, however, support routine psychosocial and sleep screening within obesity-integrated oncology care.

Chronic kidney disease has been linked to increased cancer-specific mortality in some cohorts [32]. Premuzic et al. compared histological grade and stage with glomerular filtration rate in endometrial cancer and reported an association between more advanced or aggressive disease and reduced GFR. In their cohort, the mean BMI exceeded 25 kg/m^2^, and it was higher in patients with GFR < 60 mL/min [33]. This evidence relates primarily to comorbidity burden and treatment risk rather than to cancer incidence.

Zhan et al. examined the relationship between metabolic disorders and hormonal disturbances in women’s health. The primary objective of this research was to analyse the correlation between pregnancies, menstrual history, metabolic disorders, and the incidence of endometrial cancer. Mendelian randomisation, based on genetic variants, was employed in this investigation. The cohort consisted of participants from European countries. The findings indicated a causal relationship between endometrial cancer and BMI (55.54%). Similar associations with obesity were observed at 30.37%, excluding specific obesity types, with a preference for waist circumference (27.67%), body fat percentage (17.61%), and waist-to-hip ratio (14.82%) [34].

### 5.2. Ovarian Cancer

Ovarian cancer is the second leading cause of mortality among gynaecological malignancies. Its aetiology is predominantly linked to genetic mutations or familial predisposition. BRCA gene mutations and homologous recombination deficiency (HRD) play pivotal roles in its pathogenesis. Primary tumours are categorised as low- or high-grade, and the recommended therapeutic approach depends on the stage, grade, molecular classification, and the patient’s clinical characteristics.

Ovarian cancer has also been associated with excess adiposity, although the magnitude of the association is weaker than for endometrial cancer and varies by histological subtype. Meta-analytic and consortium data support a modest BMI-related risk gradient, including increased risk for selected subtypes such as borderline, low-grade serous, mucinous and endometrioid tumours [15,18]. These findings support a cautious dose–response framework, but they do not justify the claim that morbid obesity is a uniform independent driver of all ovarian cancers.

Mechanistically, adipose tissue surrounding ovarian and peritoneal tumour deposits may contribute to tumour progression by supplying free fatty acids, adipokines, inflammatory cytokines and growth factors. This metabolic crosstalk may promote proliferation, angiogenesis, peritoneal spread and chemotherapy resistance; however, the clinical significance of this pathway varies by subtype, tumour biology and treatment context [18,35,36]. Assidi et al. reported prognostic associations of leptin expression in ovarian cancer, but no direct correlation with BMI was observed [36].

A dose–response interpretation is more appropriate than a threshold-based causal claim. Where BMI has been modelled continuously, ovarian cancer risk appears to rise modestly with each 5 kg/m^2^ increment, and cumulative exposure to overweight or obesity between young adulthood and midlife may be relevant. The signal is not uniform across ovarian cancer; it is more plausible for borderline, low-grade serous, mucinous and endometrioid tumours than for high-grade disease. Direct evidence specific to BMI ≥40 kg/m^2^ remains limited, so extrapolation from general obesity to morbid obesity should be made cautiously [15,18].

### 5.3. Cervical Cancer

Cervical cancer is predominantly HPV-driven, and HPV infection remains the essential causal pathway in the vast majority of cases. Established risk modifiers include smoking, early sexual debut, multiparity, immunosuppression and inadequate screening, while HPV vaccination is the primary preventive intervention. Obesity should therefore not be presented as replacing HPV-driven carcinogenesis.

Obesity may nevertheless affect cervical cancer prevention and care. High BMI can make pelvic examination, sampling and colposcopic visualisation more difficult, potentially reducing screening adequacy and delaying diagnosis in some settings. In treatment, morbid obesity may influence surgical selection, radiation positioning, anaesthetic planning and perioperative morbidity [10].

Cui et al. found no statistically significant Mendelian-randomisation evidence supporting obesity as a direct causal risk factor for cervical cancer [8]. This supports a cautious interpretation: the stronger clinical relevance of morbid obesity in cervical cancer may lie in detection, treatment feasibility and survival rather than in primary causation.

Experimental studies of the Kisspeptin/GPR54 pathway suggest a potential obesity-related mechanism in cervical intraepithelial neoplasia, but this remains translational and requires clinical validation before it can inform routine risk stratification [9]. These markers and pathways are therefore hypothesis-generating rather than clinically actionable biomarkers. In contrast, clinical outcome data indicate that morbid obesity may be associated with worse cervical-cancer-specific survival, even after adjustment for known prognostic factors [10].

Evidence on whether obesity modifies HPV vaccination uptake, screening attendance, sample adequacy or test performance is sparse and heterogeneous. This should be presented as a research gap rather than as an established mechanism. In current practice, the most defensible intervention is to ensure equitable access to screening, technically adequate sampling, appropriate colposcopic assessment and timely treatment planning for women with severe or morbid obesity.

## 6. Obesity-Integrated Oncological Pathway and Prehabilitation in Morbid Obesity

**Figure 4:**
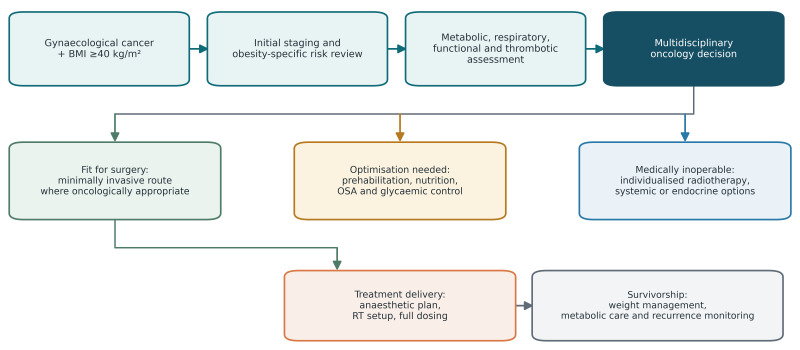
Practical obesity-integrated oncological pathway for patients with gynaecological cancer and a BMI ≥ 40 kg/m^2^. The pathway is intended as a clinical framework rather than a validated risk score.

Morbid obesity (BMI ≥ 40 kg/m^2^) poses unique challenges in gynaecological oncology that extend beyond cancer risk alone and directly affect diagnostic accuracy, perioperative safety, treatment feasibility and long-term oncological outcomes. Growing evidence supports an obesity-integrated oncological pathway that incorporates metabolic assessment, anaesthesiological risk stratification, prehabilitation, weight-appropriate systemic treatment dosing and survivorship obesity care. The proposed pathway is illustrated in Figure 4.

### 6.1. Preoperative Metabolic Assessment in Morbid Obesity

BMI alone does not fully reflect the metabolic and functional status of patients with gynaecological cancer. Therefore, a comprehensive preoperative metabolic assessment should be considered an integral part of prehabilitation before oncological treatment. Key elements include assessment of glycaemic control (HbA1c), insulin resistance indices, lipid profile, and evaluation of sarcopenic obesity using imaging-based muscle mass analysis or functional performance tests. Sarcopenic obesity, characterised by excess adiposity combined with reduced skeletal muscle mass, is increasingly recognised as a predictor of postoperative complications, impaired recovery, and worse oncological outcomes. In gynaecological cancer patients with BMI > 40 kg/m^2^, sarcopenic obesity may coexist despite high body weight and remains underdiagnosed [37].

### 6.2. Anaesthesiological Risk Stratification and OSA Screening

Morbid obesity markedly increases perioperative anaesthetic risk, largely because of the high prevalence of obstructive sleep apnoea (OSA), restrictive lung disease, and cardiovascular dysfunction [20,38]. Optimising respiratory status, meticulous airway planning, and multidisciplinary involvement of anaesthesia are essential to safe surgical management in this high-risk population [39].

### 6.3. Prehabilitation in Morbid Obesity

Prehabilitation aims to enhance physiological reserve before oncological treatment and is particularly relevant for patients with severe and morbid obesity. A multimodal prehabilitation programme should include nutritional optimisation, with adequate protein intake to counteract sarcopenia and preserve lean body mass; protein supplementation, especially for patients with reduced oral intake or sarcopenic obesity; and resistance and strength training, which have been shown to improve muscle function, insulin sensitivity and postoperative recovery [40].

### 6.4. Role of GLP-1 Receptor Agonists and Dual Incretin Therapy

Contemporary anti-obesity pharmacotherapy, including GLP-1 receptor agonists and dual incretin therapies, is a promising modality for metabolic optimisation, but its perioperative and oncological role in gynaecological cancer remains incompletely defined. These agents can reduce weight and improve insulin sensitivity, and preclinical or early translational data suggest potential interactions with endometrial cancer hormone pathways [2,37,41]. However, high-quality prospective outcome data in women with gynaecological cancer and a BMI ≥ 40 kg/m^2^ remain scarce. GLP-1 receptor agonists may delay gastric emptying and heighten aspiration risk in selected perioperative settings; recent multi-society guidance emphasises risk mitigation through symptom assessment, dietary modification, anaesthetic planning and shared decision-making rather than a uniform approach for all patients [42]. Current recommendations should therefore be interpreted as evolving expert guidance rather than conclusions from large gynaecological-oncology-specific randomised trials.

In selected patients with morbid obesity, short-term pharmacological weight reduction before definitive surgery may reduce anaesthetic risk, facilitate minimally invasive surgery and improve metabolic status. This should not delay urgent oncological treatment without multidisciplinary agreement, and the optimal timing, duration and oncological safety of pharmacological weight loss require further prospective research.

### 6.5. Timing and Oncological Relevance of Metabolic Bariatric Surgery

Although metabolic bariatric surgery is well established as an effective intervention for long-term weight reduction, its role within the oncological timeline remains complex. Available evidence suggests that bariatric surgery performed before cancer development or during long-term remission is associated with a significant reduction in cancer incidence, particularly for endometrial cancer, and may reduce cancer-related mortality [43,44,45].

There is a paucity of robust data on bariatric procedures performed after a gynaecological cancer diagnosis. Available evidence is largely observational and vulnerable to selection bias, as healthier patients, those with longer expected survival, and those in remission are more likely to be offered surgery. Potential benefits include sustained metabolic improvement, reduced oestrogen exposure and improved quality of life, but post-diagnosis metabolic surgery should be considered only after a multidisciplinary assessment of oncological urgency, treatment sequence, nutritional risk and survivorship goals.

## 7. Discussion

Endometrial cancer is the gynaecological malignancy for which the association with obesity is most extensively documented [16,46,47]. The evidence supports a strong relationship between excess adiposity and cancer incidence, but the manuscript should not imply that all obesity classes have identical biological or clinical meaning. Morbid obesity is particularly relevant to treatment feasibility, perioperative morbidity, competing mortality and survivorship care.

Obesity is a chronic condition that develops gradually, characterised by an increase in adipose tissue enriched with adipocytes. Its onset frequently occurs during childhood, influenced by limited physical activity, a diet high in fat and low in vegetables, and environmental factors. Zhu et al. documented an association between higher childhood body mass index (BMI) and ovarian cancer, irrespective of subtype (OR = 1.219, 95% CI, 1.084–1.370). The study also identified a statistically significant correlation between childhood adiposity and the incidence of endometrial cancer and its subtypes (OR = 1.417, 95% CI, 1.272–1.702). Conversely, no evidence was found to suggest a relationship between childhood obesity and the incidence of cervical cancer (*p* > 0.005). This research underscores the importance of body weight management and obesity prevention during childhood, as these measures may reduce the risk of carcinogenesis in adulthood [48].

The incidence of several early-onset cancers is rising among adults aged 20–49 years. Terashima et al. reported increasing incidence of early-onset uterine cancer in several high-income countries and rising uterine cancer mortality in selected countries [49]. These observations align with concerns about obesity, diet, physical inactivity, and environmental exposures among younger generations. However, they remain population-level associations and should not be interpreted as proof of individual causality.

Viewed through a life-course framework, childhood and adolescent adiposity may increase the likelihood of persistent adult obesity, earlier onset of insulin resistance, PCOS-related anovulation and eventual class II or III obesity. This does not prove that morbid obesity independently causes early-onset gynaecological cancer, but it strengthens the prevention message: weight management, metabolic health and reproductive-endocrine care should begin before adulthood, particularly for women at risk of endometrial cancer and selected ovarian cancer subtypes.

Polycystic ovary syndrome (PCOS) is an endocrine and metabolic disorder characterised by hyperandrogenism, ovulatory dysfunction and insulin resistance. Its relevance to endometrial cancer is mediated, in part, by anovulation, progesterone deficiency, obesity and hyperinsulinaemia [50].

Genetic and metabolic factors implicated in PCOS, including variants associated with adiposity, may contribute to increased BMI and metabolic dysfunction [50]. These observations reinforce the importance of weight management and metabolic care for adolescents and reproductive-age women, while avoiding overstatement of direct causation of cancer.

Obesity may affect human tubal ciliary beat frequency. Neville et al. reported an association between higher BMI, decreased ciliary beat frequency and the presence of gynaecological cancers (*p* = 0.024), suggesting a possible link between adiposity, tubal function and reproductive health [51]. Infertility, endometriosis, nulliparity and ovulatory dysfunction overlap with ovarian and endometrial cancer risk pathways; however, the relative contributions of infertility itself, fertility treatment and obesity remain heterogeneous and should be interpreted cautiously [52].

Opoku et al. highlight the challenges of morbid obesity in gynaecological practice. Beyond infertility and reproductive disorders, the authors note numerous psychological issues, including low self-confidence and harmful stereotypes towards individuals with obesity. These patients require more medical attention than others. Preoperative consultations with a multidisciplinary team should be considered to minimise the risk of complications during anaesthesia. The surgical technique should also be tailored to the patient’s individual needs based on the indication for surgery, comorbidities, physical condition, and anaesthetic considerations [53].

Each class of obesity may contribute to complications; however, Guzel et al.’s study revealed a 2.7-fold higher risk of mortality and a 1.7-fold higher risk of endometrial cancer recurrence. The authors encourage educating patients about lifestyle modifications and the importance of consulting dietitians and endocrinologists [19].

Large-scale epidemiological studies and meta-analyses demonstrate that the relationship between BMI and cancer risk is non-linear rather than linear, with the steepest risk gradients observed at the highest BMI levels [16].

Morbid obesity affects not only cancer incidence but also oncological outcomes. In endometrial cancer, higher BMI may be associated with poorer overall survival and increased non-cancer-related mortality, partly reflecting obesity-related comorbidities and treatment complications. This distinction is important because worse overall survival does not necessarily equate to higher cancer-specific mortality in every study.

Similarly, the landmark prospective cohort study by Calle et al. demonstrated that cancer-related mortality was most pronounced among individuals with severe and morbid obesity, particularly women with hormone-dependent malignancies [38]. Large-scale population-based analyses have corroborated that metabolic surgery is associated with a reduced risk of cancer and cancer-related mortality, particularly in women with severe obesity [44].

Furthermore, Mackenzie et al. documented a significant decrease in the incidence of endometrial cancer after metabolic surgery, thereby strengthening the notion that the oncogenic effects of morbid obesity are at least partially reversible.

Data from the Ovarian Cancer Association Consortium indicate that obesity is a risk factor for ovarian cancer, particularly at higher BMI levels, and is associated with an increased risk of borderline and low-grade serous ovarian tumours [18]. Although the overall magnitude of risk is lower than that observed for endometrial cancer, these findings suggest that excess adiposity, particularly in its most severe forms, may contribute to ovarian carcinogenesis through adipokine dysregulation, chronic inflammation, and metabolic dysfunction [18].

Morbid obesity significantly affects perioperative and adjuvant treatment outcomes. Higher BMI is linked to increased perioperative complications, including wound infection, delayed wound healing, thromboembolic events and prolonged hospital stay. Technical challenges include surgical exposure, operative duration, ventilation, positioning and radiotherapy immobilisation. Minimally invasive surgery is often advantageous when oncologically appropriate, but the surgical approach should depend on tumour stage, molecular and histological features, comorbidities, anaesthetic assessment and institutional expertise [21,22,23,24].

Systemic treatment planning should also take obesity into account. Contemporary guidance recommends avoiding empirical dose reductions solely because of obesity when cure or durable disease control is intended, while carefully monitoring toxicity and organ function [54].

Taken together, these findings support the view of morbid obesity as a distinct clinical context requiring obesity-integrated oncological pathways. They do not support a single causal model across all gynaecological cancers. The evidence is strongest for endometrial cancer incidence, more nuanced and subtype-dependent for ovarian cancer, and more clinically indirect for cervical cancer, where screening, treatment feasibility and survival effects may be more relevant than primary causation.

## 8. Future Directions

Future research should prospectively validate obesity-integrated oncological pathways in gynaecological oncology, including whether structured prehabilitation improves surgical access, perioperative morbidity, treatment completion and survivorship outcomes among patients with a BMI ≥ 40 kg/m^2^. Trials and prospective registries are particularly needed for GLP-1 receptor agonists, dual incretin therapies and multimodal prehabilitation before surgery, with explicit safeguards to prevent delays to urgent cancer treatment.

Risk models should differentiate obesity classes, including BMI ≥ 40 kg/m^2^ and BMI ≥ 50 kg/m^2^, and incorporate visceral adiposity, waist circumference, sarcopenic obesity, glycaemic status, inflammatory and metabolic markers, and life-course adiposity exposure. Additional data are needed on post-diagnosis metabolic bariatric surgery, long-term oncological outcomes, selection bias, cancer-site-specific survivorship, and interactions among obesity, HPV vaccination, screening attendance, and cervical sample adequacy.

## 9. Conclusions

Obesity is a major modifiable risk factor and clinical modifier in gynaecological oncology, but the strength and meaning of the association vary by cancer site and outcome. The strongest evidence links excess adiposity to endometrial cancer incidence via hormonal dysregulation, insulin resistance, adipokine imbalance, chronic inflammation and oxidative stress. For ovarian cancer, the association is weaker and appears more relevant to selected histological subtypes and cumulative adiposity exposure. For cervical cancer, HPV remains the dominant causal pathway; morbid obesity is more plausibly relevant to screening adequacy, treatment selection, perioperative risk and survival than to primary causation. Clinicians should currently stratify women with morbid obesity using BMI class, waist circumference or visceral adiposity, diabetes and insulin-resistance markers, OSA, sarcopenic obesity, renal and cardiovascular comorbidity, functional status and cancer-site-specific diagnostic and treatment requirements. Biomarker evidence remains hypothesis-generating and should not be used for routine obesity-based risk stratification. Morbid obesity should therefore be addressed through structured risk stratification, prehabilitation, anaesthesiological planning, weight-appropriate systemic-treatment dosing and survivorship obesity care. The available evidence does not demonstrate that morbid obesity is an independent driver of all gynaecological cancers, and future studies should validate obesity-integrated pathways prospectively.

## Figures and Tables

**Table 1:** Body mass index categories used to classify weight status and obesity.

BMI Category	Minimum BMI [kg/m^2^]	Maximum BMI [kg/m^2^]
Underweight	No lower limit	<18.5
Normal weight	18.5	24.9
Overweight	25.0	29.9
Obesity class I	30.0	34.9
Obesity class II (severe obesity)	35.0	39.9
Obesity class III (morbid obesity)	≥40.0	No upper limit

**Table 2:** Summary of key evidence linking obesity, morbid obesity and gynaecological cancer risk, outcomes and management.

Evidence/Source	Design/Population	Cancer Site	Obesity Measure	Main Finding	Relevance to Morbid Obesity
IARC/body-fatness evidence [4]	Expert review of carcinogenicity evidence	Multiple cancers, including female-specific cancers	Excess body fatness	Excess adiposity is causally linked to several cancers; the strongest female-specific association is endometrial cancer.	Supports obesity as a major modifiable risk factor but not a uniform driver of all gynaecological cancers.
Renehan et al. [15]	Systematic review and meta-analysis of prospective studies	Multiple cancer sites	BMI increments	Higher BMI is associated with increased cancer incidence; risk magnitude differs by cancer site.	Supports a dose-related risk framework; does not isolate BMI ≥ 40 kg/m^2^ in all cancer sites.
Aune et al. [16]	Dose-response meta-analysis	Endometrial cancer	BMI and anthropometric factors	Endometrial cancer risk increases substantially with higher adiposity measures.	Highly relevant; strengthens the endometrial-cancer focus.
Kang et al. [17]	Nationwide retrospective cohort	Gynaecological malignancies	Obesity indices	Obesity-related indices show different relationships across gynaecological malignancies.	Supports separating BMI category, visceral adiposity and cancer site.
Zhang et al. [11]	NHANES cross-sectional analysis	Endometrial, ovarian and cervical cancers	AVAI, BMI, waist circumference, triglycerides, HDL	Higher visceral adiposity index was associated with gynaecological cancer overall, with site-specific differences.	Useful for visceral-adiposity discussion; not sufficient for causal inference.
Olsen et al. [18]	Ovarian Cancer Association Consortium analysis	Ovarian cancer subtypes	BMI	Associations differ by ovarian cancer histotype, with stronger signals for borderline and selected low-grade/subtype-specific tumours.	Supports a cautious, subtype-dependent interpretation for ovarian cancer.
Guzel et al. [19]	Clinical outcome study	Endometrial cancer	Morbid obesity	Morbid obesity was associated with worse survival and recurrence-related outcomes.	Directly relevant to BMI ≥ 40 kg/m^2^ and survivorship/recurrence interpretation.
Gunderson et al. and surgical literature [20,21,22,23,24]	Clinical and surgical outcome studies/guidelines	Gynaecological cancer management	BMI and morbid obesity	Higher BMI complicates surgical staging, minimally invasive access, anaesthesia, radiotherapy planning and postoperative recovery.	Supports obesity-integrated perioperative and prehabilitation pathways.

**Table 3:** Pragmatic interpretation of obesity-related evidence by cancer site and outcome domain.

Cancer Site	Evidence Interpretation	Morbid-Obesity Relevance	Current Stratification
Endometrial cancer	Strongest incidence signal; dose-response across BMI and anthropometric measures.	Class III obesity is especially relevant to recurrence, overall survival, perioperative morbidity and competing mortality.	Use BMI class plus waist/visceral adiposity, diabetes/insulin resistance, PCOS/anovulation, OSA, renal/cardiovascular disease and functional status.
Ovarian cancer	Modest, histotype-dependent association; stronger signals for borderline, low-grade serous, mucinous and endometrioid tumours.	Direct BMI ≥ 40 kg/m^2^ evidence is limited; prognosis remains dominated by stage, histotype and molecular profile.	Do not use BMI alone; combine adiposity duration, metabolic syndrome, sarcopenia/functional reserve and tumour histotype.
Cervical cancer	HPV-driven disease; obesity is not established as a primary aetiological driver.	Morbid obesity may affect screening adequacy, treatment feasibility and disease-specific survival.	Treat obesity as a management modifier: ensure screening/vaccination access, sample adequacy, radiotherapy setup and perioperative risk review.

**Table 4:** Systematisation of obesity-induced mechanisms involved in endometrial carcinogenesis.

Mechanistic Domain	Obesity-Related Alteration	Endometrial Effect	Clinical Interpretation
Hormonal dysregulation	Adipose-tissue aromatisation, lower SHBG, higher bioavailable oestrogens	Unopposed oestrogenic stimulation of endometrium	Most relevant to endometrioid/type I endometrial cancer.
Insulin resistance	Hyperinsulinaemia, IGF-1 pathway activation	Increased proliferation and reduced apoptosis	Links obesity, diabetes and endometrial cancer risk.
Adipokines and inflammation	High leptin, lower adiponectin signalling, TNF-α and IL-6 activity	Angiogenesis, mitogenic signalling and inflammatory microenvironment	Supports obesity as a biological risk factor but not a site-uniform driver.
Oxidative stress and genomic instability	Reactive oxygen species and chronic cellular stress	DNA damage and potential acceleration of tumour evolution, especially in susceptible tissue	Relevant to mismatch-repair-deficient pathways; evidence remains mechanistic.
Microbiome dysbiosis	Obesity-associated gut and local microbial changes	Possible immune and inflammatory modulation	Emerging hypothesis; not yet ready for routine clinical risk stratification.

## Data Availability

No new data were created or analyzed in this study. Data sharing is not applicable to this article.

## Abbreviations

The following abbreviations are used in this manuscript:

LVSI	Lymphovascular space invasion
BMI	Body mass index
EC	Endometrial cancer
OC	Ovarian cancer
CC	Cervical cancer
AVAI	Age-adjusted visceral adiposity index
GLP-1	Glucagon-like peptide-1
HDL	High-density lipoprotein
HPV	Human papillomavirus
HRD	Homologous recombination deficiency
IGF-1	Insulin-like growth factor 1
OSA	Obstructive sleep apnoea
PCOS	Polycystic ovary syndrome
SHBG	Sex hormone-binding globulin
STC-1	Stanniocalcin 1
WC	Waist circumference
